# Exploration of serum biomarkers in dogs with malignant melanoma receiving anti-PD-L1 therapy and potential of COX-2 inhibition for combination therapy

**DOI:** 10.1038/s41598-022-13484-8

**Published:** 2022-06-03

**Authors:** Naoya Maekawa, Satoru Konnai, Yumie Asano, Yamato Sajiki, Tatsuya Deguchi, Tomohiro Okagawa, Kei Watari, Hiroto Takeuchi, Satoshi Takagi, Kenji Hosoya, Sangho Kim, Hiroshi Ohta, Yukinari Kato, Yasuhiko Suzuki, Shiro Murata, Kazuhiko Ohashi

**Affiliations:** 1 Department of Advanced Pharmaceutics, Faculty of Veterinary Medicine, Hokkaido University, Sapporo, Japan; 2 Department of Disease Control, Faculty of Veterinary Medicine, Hokkaido University, Sapporo, Japan; 3 Veterinary Teaching Hospital, Faculty of Veterinary Medicine, Hokkaido University, Sapporo, Japan; 4 Department of Veterinary Surgery 1, School of Veterinary Medicine, Azabu University, Sagamihara, Japan; 5 Department of Antibody Drug Development, Tohoku University Graduate School of Medicine, Sendai, Japan; 6 Department of Molecular Pharmacology, Tohoku University Graduate School of Medicine, Sendai, Japan; 7 International Institute for Zoonosis Control, Hokkaido University, Sapporo, Japan; 8 Global Station for Zoonosis Control, Global Institution for Collaborative Research and Education (GI-CoRE), Hokkaido University, Sapporo, Japan

**Keywords:** Cancer immunotherapy, Tumour immunology

## Abstract

Immune checkpoint inhibitors (ICIs) such as anti-PD-L1 antibodies are widely used to treat human cancers, and growing evidence suggests that ICIs are promising treatments for canine malignancies. However, only some canine oral malignant melanoma (OMM) cases respond to ICIs. To explore biomarkers predictive of survival in dogs with pulmonary metastatic OMM receiving the anti-PD-L1 antibody c4G12 (*n* = 27), serum concentrations of prostaglandin E2 (PGE_2_), cytokines, chemokines, and growth factors were measured prior to treatment initiation. Among 12 factors tested, PGE_2_, interleukin (IL)-12p40, IL-8, monocyte chemotactic protein-1 (MCP-1), and stem cell factor (SCF) were higher in OMM dogs compared to healthy dogs (*n* = 8). Further, lower baseline serum PGE_2_, MCP-1, and vascular endothelial growth factor (VEGF)-A concentrations as well as higher IL-2, IL-12, and SCF concentrations predicted prolonged overall survival. These observations suggest that PGE_2_ confers resistance against anti-PD-L1 therapy through immunosuppression and thus is a candidate target for combination therapy. Indeed, PGE_2_ suppressed IL-2 and interferon (IFN)-γ production by stimulated canine peripheral blood mononuclear cells (PBMCs), while inhibition of PGE_2_ biosynthesis using the COX-2 inhibitor meloxicam in combination with c4G12 enhanced Th1 cytokine production by PBMCs. Thus, serum PGE_2_ may be predictive of c4G12 treatment response, and concomitant use of COX-2 inhibitors may enhance ICI antitumor efficacy.

## Introduction

Cancer has become a major cause of death in domesticated dogs due to gains in lifespan. Like human cancers, canine cancers are usually treated by surgical excision, radiation, chemotherapy, or a combination of these measures. In addition, new treatment modalities are being developed to provide better veterinary care for canines. Immunotherapy is one promising strategy because the therapeutic effect is expected to be systemic but still cancer-specific. In humans, antibodies that inhibit immune checkpoint molecules, such as programmed cell death 1 (PD-1) and PD-ligand 1 (PD-L1), have demonstrated robust antitumor efficacies with acceptable safety profiles for various cancer types^1–3^. PD-1 is an inhibitory receptor that suppresses the effector functions of activated T cells. Its ligand, PD-L1, is often overexpressed in tumor cells, suggesting that the PD-1/PD-L1 pathway is a major mechanism for immune evasion by tumors^4,5^. In dogs, PD-1 expression is upregulated in lymphocytes infiltrating oral malignant melanoma (OMM), and PD-L1 is detected in tumor cells of various malignant cancers including OMM and osteosarcoma^6–16^. A recently developed canine chimeric anti-PD-L1 antibody (c4G12) demonstrated promising antitumor activity in dogs with OMM and the survival benefit was strongly suggested^11,16^. However, the majority of dogs with OMM did not respond to c4G12^16^, suggesting that other factors may limit ICI efficacy. These findings emphasize the need for further study on predictive biomarkers that can distinguish the subpopulation most likely to benefit from ICI therapy. In addition, studies are required to elucidate mechanisms limiting ICI antitumor activity and thereby identify possible targets for combination therapy.

Peripheral blood or serum/plasma biomarkers are considered preferable because sampling is easy and less invasive. To date, several serum factors have been identified that are predictive of ICI benefit among human cancer patients, including C reactive protein (CRP), interleukin (IL)-6, soluble PD-L1, and various cytokines and chemokines^17–21^. We previously reported that high baseline CRP in plasma was associated with poorer overall survival (OS) in dogs with pulmonary metastatic OMM receiving c4G12 treatment^16^; however, the predictive values of cytokines, chemokines, and other factors have not been investigated in canine cancer. Hence, to explore additional predictive biomarkers for ICI efficacy in dogs with OMM, we first measured serum concentrations of multiple immune modulators [prostaglandin E2 (PGE_2_), the cytokines interferon-γ (IFN-γ), IL-2, IL-6, IL-10, IL-12p40, and tumor necrosis factor-α (TNF-α), the chemokines IL-8 and monocyte chemotactic protein 1 (MCP-1), and the growth factors nerve growth factor-β (NGF-β), stem cell factor (SCF), and vascular endothelial growth factor-A (VEGF-A)] in healthy controls and dogs with pulmonary metastatic OMM prior to c4G12 treatment. The associations of serum concentrations with OS were assessed by univariate analysis. Next, we examined the clinical relevance and functions of PGE_2_ in canine cancers because PGE_2_ is a known suppressor of T cell responses in humans through binding to E prostanoid 2 (EP2) and EP4 receptors^22^ and inhibitors of its biosynthetic enzyme cyclooxygenase-2 (COX-2) are available for canine diseases with well-known efficacy and safety profiles. Overexpression of COX-2 has been reported in various canine cancers including transitional cell carcinoma, squamous cell carcinoma, and mammary cancer^23^, and the selective COX-2 inhibitor piroxicam^24^ has demonstrated clinical benefit against these canine cancers^25–27^. Recent preclinical studies using mouse cancer models have demonstrated that COX inhibition enhances immunotherapeutic efficacy of anti-PD-1 therapy^28,29^, however, suppressive functions of PGE_2_ in the canine immune system and contributions to cancer immune evasion remain unclear. Therefore, to assess the potential of COX-2 inhibition plus anti-PD-L1 antibody as combination therapy, we first confirmed *COX2* mRNA expression and PGE_2_ upregulation in canine cancers, and then examined the immunosuppressive effects of PGE_2_ in cultures of canine peripheral blood mononuclear cells (PBMCs). Finally, the combinational effect of the selective COX-2 inhibitor meloxicam plus anti-PD-L1 antibody was investigated in PBMCs for possible applicability to canine cancer treatment.

## Materials and methods

### Canine samples

Animal care and use protocols were approved by the Institutional Animal Care and Use Committee of Hokkaido University (Approval number: 15–0149). All experiments were performed in accordance with relevant guidelines and regulations of the Faculty of Veterinary Medicine, Hokkaido University, which is fully accredited by the Association for Assessment and Accreditation of Laboratory Animal Care International. The use of animals throughout the clinical study was approved by the ethics committee, Faculty of Veterinary Medicine, Hokkaido University. The reporting of the animal experiment in this study follows the recommendations in the ARRIVE guidelines. Serum samples from pulmonary metastatic OMM dogs (stage IV as defined by TNM-based staging^30^, *n* = 27) treated with the canine chimeric anti-PD-L1 antibody c4G12^11^ at the Veterinary Teaching Hospital of Hokkaido University were collected at baseline (on the first day of immunotherapy). The detailed dosage and duration of c4G12 therapy and the baseline characteristics of treated dogs were described elsewhere^16^. Plasma samples were obtained from tumor-bearing dogs (various tumor types, *n* = 21) examined at the Veterinary Teaching Hospital of Hokkaido University. For healthy dog samples, serum, plasma, and heparinized whole blood were collected from clinically healthy beagles housed at the Experimental Animal Facility, Faculty of Veterinary Medicine, Hokkaido University. Blood sampling was performed without anesthesia. Before sample collection, informed consent was obtained from the dogs’ owners.

### Multiplex immunoassay and enzyme-linked immunosorbent assay (ELISA)

Serum concentrations of IFN-γ, IL-2, IL-6, IL-10, IL-12/IL-23p40, TNF-α, IL-8, MCP-1, NGF-β, SCF, and VEGF-A were quantified by bead-based multiplex immunoassays using Cytokine/Chemokine/Growth Factor 11-Plex Canine ProcartaPlex Panel 1 (Thermo Fisher Scientific, Waltham, MA) and the Luminex 200 System (Luminex, Austin, Texas). Data were analyzed using Bio-Plex Manager version 6.1 (Bio-Rad, Hercules, CA). PGE_2_ concentrations in plasma, serum and culture supernatant were quantified using the Prostaglandin E2 Express ELISA Kit (Cayman Chemical, Ann Arbor, MI). IL-2 concentrations in culture supernatant were quantified using the Canine IL-2 DuoSet ELISA kit, and IFN-γ concentrations using the Canine IFN-γ DuoSet ELISA kit (both from R&D Systems, Minneapolis, MN). The optical density was measured using the MTP-900 microplate reader (Corona Electric, Ibaraki, Japan). A heat map showing relative serum levels of each factor was generated using Heatmapper, a web-based tool previously described by Babicki et al*.* (http://www.heatmapper.ca/)^31^. Values below the lower limit of quantification (LLOQ) are marked as missing data.

### Cell culture

The canine melanoma cell lines CMeC, LMeC, CMM-1, and CMM-2^32,33^, were cultured as described previously^6^. The canine osteosarcoma cell lines POS^34^ and HMPOS^35^ were cultured in RPMI 1640 medium (Sigma-Aldrich, St. Louis, MO) supplemented with 10% fetal calf serum (FCS), 2 mM L-glutamine, 200 μg/mL streptomycin, and 200 U/mL penicillin (Thermo Fisher Scientific) at 37 °C under a 5% CO_2_ atmosphere. Canine PBMCs were purified from heparinized blood obtained from healthy beagles by density gradient centrifugation on Percoll (GE Healthcare UK, Buckinghamshire, UK) and cultured as described previously^6^. PBMCs were stimulated with 5 μg/mL Staphylococcal Enterotoxin B (SEB) (Sigma-Aldrich) and 1 μg/mL anti-canine CD28 antibody (eBioscience, San Diego, CA). In some experiments, cells were co-treated with 5 μM meloxicam (Sigma-Aldrich), 2.5 μM Prostaglandin E2 (Cayman Chemical), and/or 20 μg/mL canine chimeric anti-PD-L1 antibody c4G12^11^ as indicated. The same concentrations of DMSO (Nacalai Tesque, Kyoto, Japan) and dog IgG (Jackson ImmunoResearch, West Grove, PA) were used as negative controls.

### Reverse transcription quantitative polymerase chain reaction (RT-qPCR)

Total RNA was extracted from canine cancer cell lines using TRI reagent (Molecular Research Center, Cincinnati, OH) and residual genomic DNA was digested by DNase I (Thermo Fisher Scientific) treatment. cDNA was synthesized from 1 μg of RNA using PrimeScript RTase (TaKaRa, Otsu, Japan) and oligo-dT primer in the presence of 20 U RNase inhibitor (Promega, Madison, WI). For estimation of *COX2* expression (GenBank accession number NM_001003354.1), qPCR was conducted in a reaction mixture including the primers 5′-AAG CTT CGA TTG ACC AGA GCA G-3′ and 5′-TCA CCA TAA AGG GCC TCC AAC-3′, 1 μL cDNA, and SYBR Premix DimerEraser (TaKaRa) using the LightCycler480 System II (Roche Diagnostics, Mannheim, Germany). *HPRT1* (GenBank accession number AY283372.1) was used as an internal control gene^13^. Relative *COX2* mRNA expression level was calculated relative to *HPRT1* expression.

### Statistical analysis

Paired data were compared by Wilcoxon signed rank test, unpaired data by Mann–Whitney U test, and > 2 group data by Steel–Dwass test. OS was compared between groups stratified according to the optimal cutoff value for each measured factor (high group > cutoff and low group ≤ cutoff) by Kaplan–Meier analysis. The optimal cutoff value for each factor was defined as that yielding the most significant split by log-rank test as described by Budczies et al*.* (Cutoff Finder)^36^. OS was defined as time in days from the first dose of c4G12 until death. All deaths except for one were considered tumor-related and euthanasia was performed in one dog due to disease progression^16^ (Supplementary Table 1). Receiver operating characteristic (ROC) curves for each serum factor were generated in relation to longer survival (OS > median) or tumor response as evidenced by diagnostic imaging^16^. Youden’s index was used to determine the cutoff value for calculation of sensitivity and specificity. Statistical analysis was performed using Fisher's exact test. All tests were performed using EZR statistical software^37^, and *P* < 0.05 (two-tailed) was considered statistically significant.

## Results

### Multiple serum factors are upregulated in dogs with pulmonary metastatic OMM

**Figure 1:**
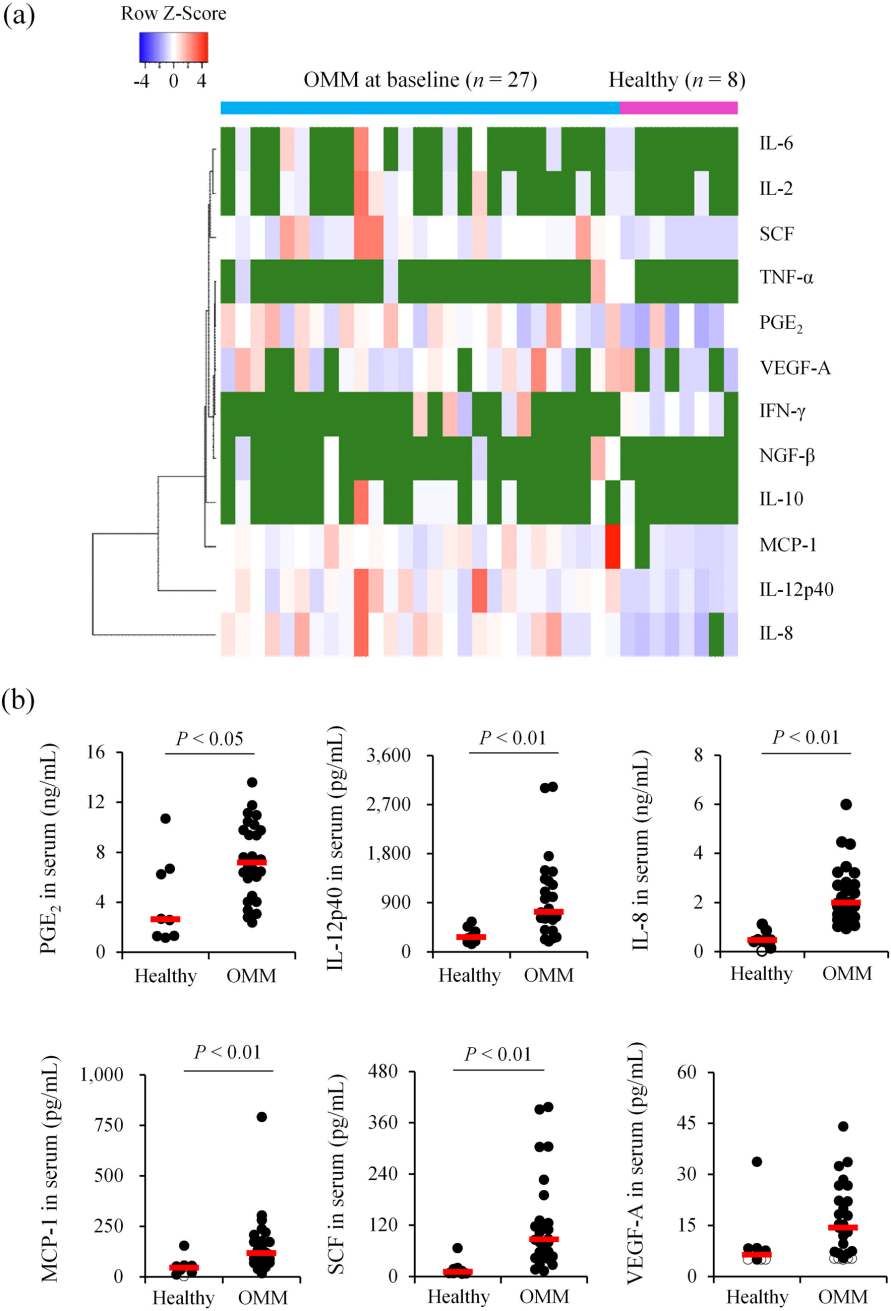
Serum concentrations of several immune modulators are elevated in dogs with pulmonary metastatic oral malignant melanoma (OMM). (**a**) Heat map depicting the serum concentrations of each measured factor (as Z-Scores) in dogs with OMM prior to treatment (*n* = 27) and in healthy dogs (*n* = 8). Missing values (below the lower limit of quantitation [LLOQ]) are shown in dark green. (**b**) Comparison of serum concentration between OMM and healthy dogs. Red bars indicate the median values. Values below LLOQ are shown as open circles. Statistical analysis was performed using Mann–Whitney U test.

To examine whether serum levels of PGE_2_, various cytokines, chemokines, and growth factors are of clinical relevance in canine OMM, we first compared serum concentrations between healthy controls (*n* = 8) and dogs with pulmonary metastatic OMM (*n* = 27 among a cohort of 29 dogs^16^) before the first dose of c4G12 using ELISA and a bead-based immunoassay panel (Fig. 1a). The baseline characteristics of c4G12-treated dogs, treatment outcome, and serum concentration of each factor are shown in Supplementary Tables 1 and 2. Among these 12 factors tested, PGE_2_, IL-12p40, IL-8, MCP-1, and SCF were quantifiable in most dogs and the concentrations were significantly higher in OMM dogs than healthy control dogs (Fig. 1b). In addition, VEGF-A was measurable in 22 OMM dogs (81.5%) and five healthy dogs (62.5%), and serum concentration tended to be higher in OMM dogs (Fig. 1b). The serum concentrations of the other six measured factors were below the LLOQ in too many animals to allow statistical comparison.

### Baseline concentrations of serum factors are associated with overall survival in dogs with pulmonary metastatic OMM receiving anti-PD-L1 antibody therapy

**Figure 2:**
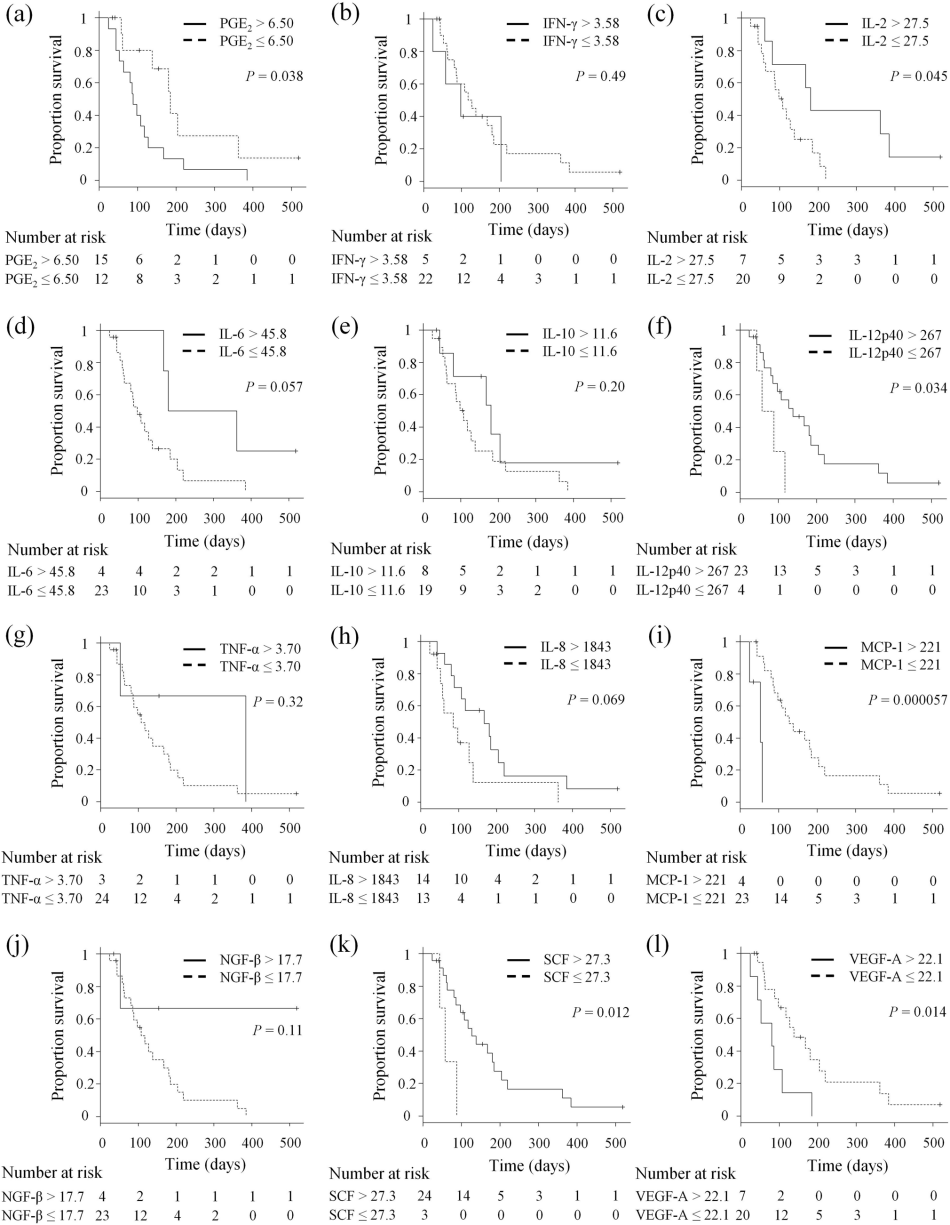
Baseline serum concentrations of several immune modulators are associated with overall survival (OS) in dogs with pulmonary metastatic OMM receiving c4G12 therapy. Dogs were dichotomized into subgroups based on the indicated cutoff value for (**a**) Prostaglandin E2 (PGE_2_), (**b**) interferon-γ (IFN-γ), (**c**) interleukin (IL)-2, (**d**) IL-6, (**e**) IL-10, (**f**) IL-12p40, (**g**) tumor necrosis factor-α (TNF-α), (**h**) IL-8, (**i**) monocyte chemotactic protein 1 (MCP-1), (**j**) nerve growth factor-β (NGF-β), (**k**) stem cell factor (SCF), and (**l**) vascular endothelial growth factor-A (VEGF-A), and Kaplan–Meier curves were constructed comparing each corresponding subgroup (high group > cutoff vs. low group ≤ cutoff). Statistical analysis was performed using log-rank test.

To explore serum biomarkers predictive of clinical outcome among dogs with OMM receiving anti-PD-L1 therapy, the OMM group was dichotomized according to serum concentration of each factor and the OS was compared between high (> cutoff) and low (≤ cutoff) groups by Kaplan–Meier analysis. Higher serum levels of PGE_2_, MCP-1, and VEGF-A were associated with worse OS in animals receiving c4G12 therapy (*P* = 0.038, 5.7 × 10^−5^, and 0.014, respectively; Fig. 2). In contrast, higher serum IL-2, IL-12p40, and SCF were correlated with improved OS (*P* = 0.045, 0.034, and 0.012, respectively; Fig. 2). There were no significant associations between other serum factors (IFN-γ, IL-6, IL-10, TNF-α, IL-8, and NGF-β) and OS (*P* > 0.05, Fig. 2). To help interpret the predictive power of each serum factor, ROC analysis was performed in relation to longer survival (OS > median) or tumor response. Area under the curve (AUC), sensitivity, and specificity for each factor are shown in Tables 1 and 2. Partially consistent with the Kaplan–Meier analysis, PGE_2_ and IL-6 were significant predictors of longer survival (*P* = 0.033 and 0.031, respectively), whereas IL-2 and IL-6 predicted tumor response to c4G12 treatment (*P* = 0.030 and 0.013, respectively).

**Table 1:** ROC analysis of each serum factor in relation to longer survival.

	AUC (95% CI)	Cutoff*	Sensitivity	Specificity	*P* value**
PGE_2_	0.727 (0.501–0.954)	6.5	0.636	0.846	0.033
IFN-γ	0.566 (0.416–0.717)	< LLOQ	0.231	0.909	0.596
IL-2	0.626 (0.398–0.854)	17.7	0.545	0.769	0.206
IL-6	0.535 (0.299–0.771)	142.4	0.364	1.000	0.031
IL-10	0.622 (0.412–0.833)	18.4	0.455	0.846	0.182
IL-12p40	0.587 (0.341–0.834)	388.5	1.000	0.308	0.098
TNF-α	0.521 (0.362–0.680)	4.7	0.182	0.923	0.576
IL-8	0.573 (0.329–0.818)	1980.8	0.727	0.538	0.240
MCP-1	0.664 (0.440–0.889)	48.7	0.273	1.000	0.082
NGF-β	0.521 (0.362–0.680)	28.0	0.182	0.923	0.576
SCF	0.741 (0.531–0.951)	87.7	0.727	0.692	0.100
VEGF-A	0.594 (0.353–0.835)	22.1	0.909	0.462	0.211

The sensitivity and specificity of each factor to predict longer survival (OS > median) are shown.

AUC, area under the curve; CI, confidence interval; LLOQ, lower limit of quantification.

*Cutoffs were determined by calculating the Youden's index.

**Fisher's exact test.

**Table 2:** ROC analysis of each serum factor in relation to tumor response.

	AUC (95% CI)	Cutoff*	Sensitivity	Specificity	*P* value**
PGE_2_	0.618 (0.285–0.951)	3.1	0.400	0.955	0.628
IFN-γ	0.614 (0.524–0.703)	< LLOQ	1.000	0.227	0.547
IL-2	0.818 (0.560–1.000)	33.0	0.800	0.864	0.030
IL-6	0.727 (0.414–1.000)	142.4	0.600	0.955	0.013
IL-10	0.691 (0.391–0.991)	84.6	0.400	1.000	0.136
IL-12p40	0.727 (0.470–0.985)	651.9	1.000	0.455	0.561
TNF-α	0.664 (0.407–0.920)	4.7	0.400	0.955	0.079
IL-8	0.618 (0.317–0.920)	2007.0	0.800	0.591	0.326
MCP-1	0.718 (0.524–0.913)	115.2	1.000	0.591	0.474
NGF-β	0.641 (0.380–0.902)	28.0	0.400	0.909	0.144
SCF	0.773 (0.520–1.000)	125.0	0.800	0.818	0.165
VEGF-A	0.500 (0.228–0.772)	17.9	0.600	0.636	1.000

The sensitivity and specificity of each factor to predict tumor response are shown.

AUC, area under the curve; CI, confidence interval; LLOQ, lower limit of quantification.

*Cutoffs were determined by calculating the Youden's index.

**Fisher's exact test.

### The COX-2/PGE_2_ axis is a potential immune evasion mechanism in canine cancers

**Figure 3:**
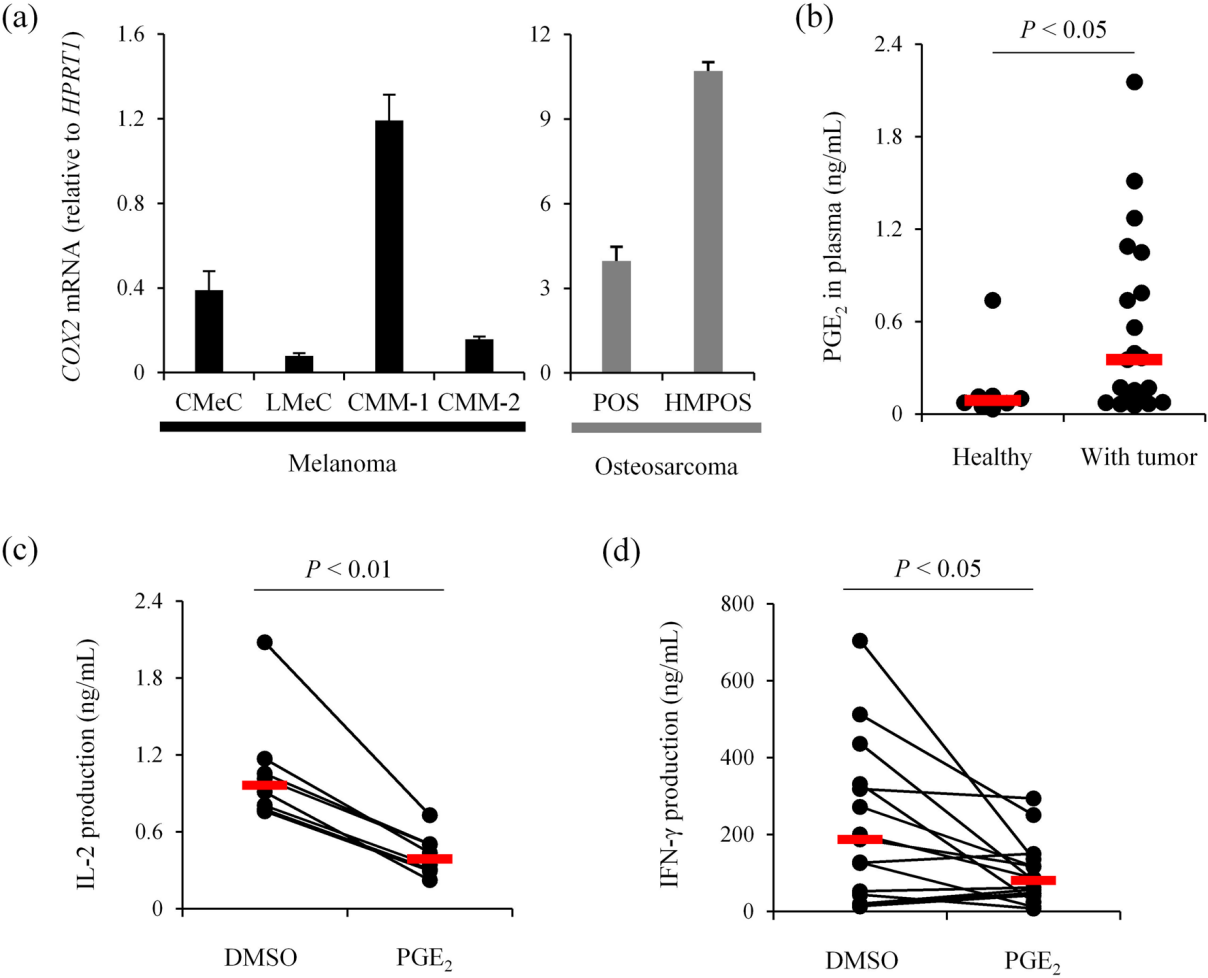
PGE_2_ is a potential immunosuppressive factor in canine cancer. (**a**) *COX2* mRNA expression in canine cancer cell lines. Total RNA was extracted from each cancer cell line and *COX2* mRNA expression was measured by RT-qPCR. *HPRT1* expression was used as an internal control. The mean values of triplicate measurement are shown. Error bars indicate the standard deviation (SD). (**b**) Plasma PGE_2_ concentration in dogs with tumors. Plasma samples were collected from healthy (*n* = 8) and tumor-bearing dogs (*n* = 21). Red bars indicate the median values. Statistical analysis was performed using Mann–Whitney U test. (**c**,**d**) Suppression of cytokine production from canine peripheral blood mononuclear cells (PMBCs) by PGE_2_. Canine PBMCs were cultured for 3 d with or without 2.5 μM PGE_2_, and concentrations of (**c**) IL-2 (*n* = 7) and (**d**) IFN-γ (*n* = 15) in the supernatant were measured by ELISA. Red bars indicate the median values. Statistical analysis was performed using Wilcoxon signed rank test.

Based on the significant association between elevated serum PGE_2_ and shorter OS in OMM dogs receiving c4G12 therapy, we speculated that the COX-2/PGE_2_ pathway suppresses antitumor immunity and thus confers resistance against anti-PD-L1 therapy. Consistent with previous studies demonstrating high COX-2 expression in canine cancers including melanoma^23^, all canine melanoma cell lines (CMeC, LMeC, CMM-1, and CMM-2) and osteosarcoma cell lines (POS and HMPOS) examined had detectable *COX2* mRNA expression (Fig. 3a). To verify that peripheral blood PGE_2_ levels are elevated in dogs with tumor, plasma concentration of PGE_2_ was measured in another cohort of dogs with various tumor (*n* = 21; see Supplementary Table 3 for details). Indeed, plasma PGE_2_ concentration was higher in tumor dogs compared to a healthy control group (*n* = 8; Fig. 3b), suggesting that PGE_2_ is generally upregulated in canine tumor, possibly through aberrant expression of COX-2 in tumor tissues.

To investigate the immunosuppressive potential of PGE_2_ in dogs, PBMCs from healthy dogs were cultured with the nonspecific T cell stimulators SEB and anti-CD28 antibody for 3 d in the presence of PGE_2_. PGE_2_ treatment significantly reduced IL-2 and IFN-γ production by stimulated canine PBMCs (Fig. 3c,d), suggesting that PGE_2_ is a potent suppressor of canine T cell responses.

### Combined COX-2 inhibition and anti-PD-L1 antibody treatment enhances cytokine production from canine PBMCs

**Figure 4:**
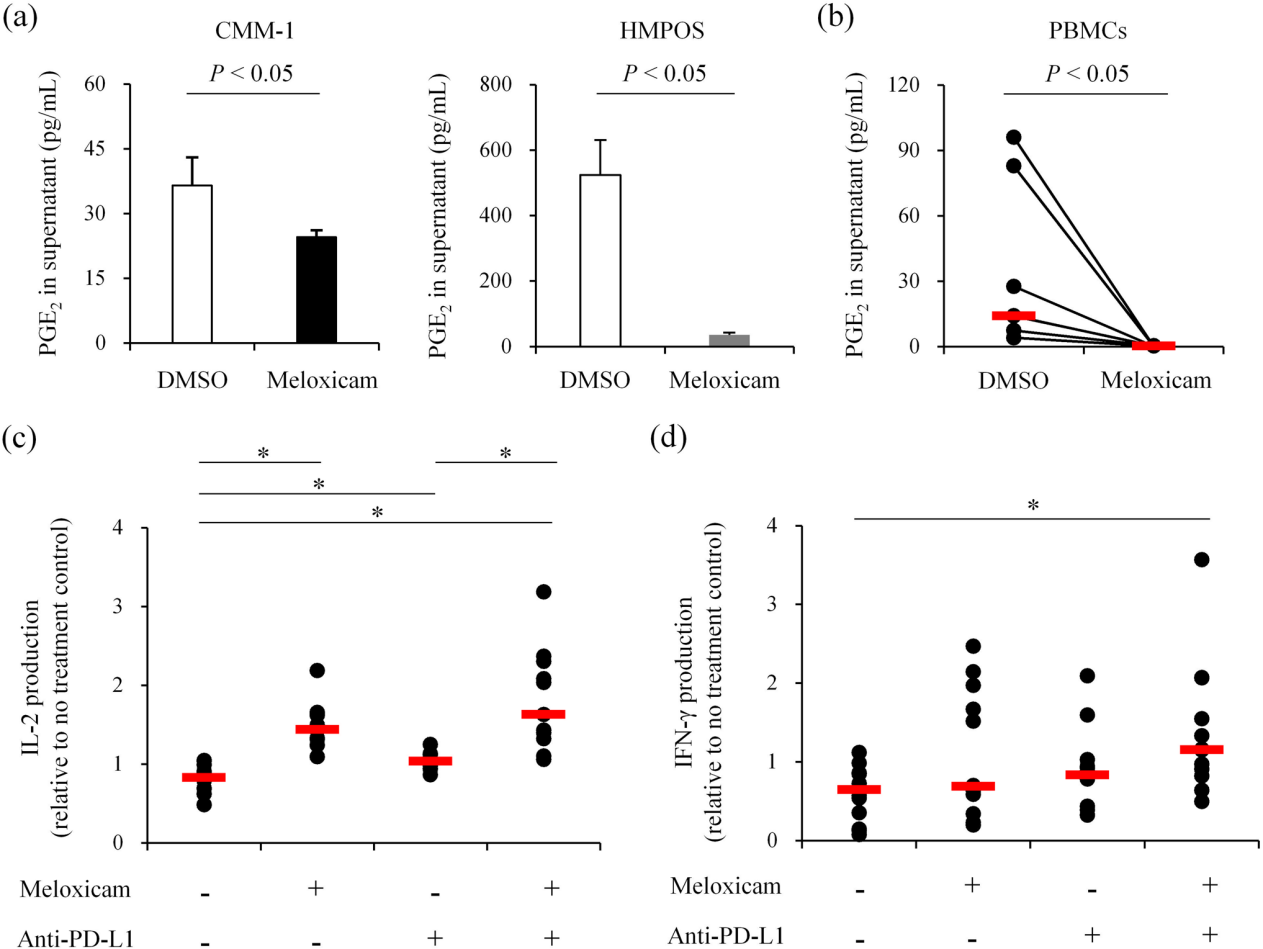
The cyclooxygenase-2 (COX-2) inhibitor meloxicam reduces PGE_2_ production and enhances cytokine production by canine PBMCs with or without anti-programmed death ligand 1 (PD-L1) antibody treatment. (**a**,**b**) Suppression of PGE_2_ production by the COX-2 inhibitor meloxicam. Cells were cultured for 3 d with 5 μM meloxicam, and PGE_2_ concentration in the supernatant was measured by ELISA. (**a**) PGE_2_ production from canine cancer cell lines CMM-1 and HMPOS. Mean values of triplicate measurement are shown. Error bars indicate the SD. Statistical analysis was performed using Mann–Whitney U test. (**b**) PGE_2_ production from canine PBMCs (*n* = 7). Red bars indicate the median values. Statistical analysis was performed using Wilcoxon signed rank test. (**c**,**d**) Enhancement of cytokine production from canine PBMCs by meloxicam treatment. Canine PBMCs were cultured for 3 d with 5 μM meloxicam and/or 20 μg/mL anti-PD-L1 antibody c4G12, and (**c**) IL-2 (*n* = 11) and (**d**) IFN-γ (*n* = 15) concentrations in the supernatant were measured by ELISA. Each point represents the relative cytokine concentration compared to no treatment control prepared from the same individual. Red bars indicate the median values. Statistical analysis was performed using Steel–Dwass test (*P < 0.05).

The selective COX-2 inhibitor meloxicam is used routinely for anti-inflammation and analgesia in veterinary practice. To assess whether PGE_2_ production from canine cancer cells is COX-2 dependent, the CMM-1 and HMPOS cell lines, both with relatively high baseline *COX2* expression (Fig. 3a), were treated with meloxicam. PGE_2_ concentrations in the culture supernatant were significantly reduced after 3 d of meloxicam treatment compared to untreated control cells (Fig. 4a). In the tumor microenvironment, infiltrating immune cells can be an additional source of PGE_2_^38^. Indeed, canine PBMCs stimulated in vitro with SEB and anti-CD28 antibody produced detectable amount of PGE_2_ that was significantly reduced by meloxicam (Fig. 4b).

To evaluate the immunostimulatory potential of meloxicam, PBMCs were treated with meloxicam alone or in combination with the anti-PD-L1 antibody c4G12. Both meloxicam and anti-PD-L1 antibody enhanced IL-2 production from stimulated canine PBMCs when applied individually, whereas the increase in IFN-γ production was not statistically significant. However, combination treatment further enhanced IL-2 production and significantly increased IFN-γ production (Fig. 4c,d), suggesting that dual blockade of PD-1/PD-L1 and COX-2/PGE_2_ pathways is a promising strategy to enhance antitumor immune responses in dogs.

## Discussion

Immunotherapy is now recognized as the “fourth pillar of human cancer treatment”, with clinical importance equivalent to surgery, radiotherapy, and traditional chemotherapy. The applications of cancer immunotherapy are spreading, particularly as combination treatment, including with molecular-targeted drugs. In veterinary medicine, a few clinical studies, including from our group, have examined the efficacy of immune checkpoint blockade for dogs with cancer; however, as in human cancer^2,3,39^, only a subset of dogs responded well to ICI therapy^11,16,40^. The identification of biomarkers predictive of ICI response will not only optimize treatment efficacy but may also provide clues to the mechanisms underlying tumor immune evasion in dogs as well as humans. Indeed, dogs are an excellent animal model for human cancer research because they develop naturally occurring tumors that share many features with the corresponding human cancers, such as invasiveness and metastatic propensity, molecular aberrations, heterogeneity, and response/resistance to treatment^41^. As dogs are immunocompetent and genetically outbred, they could be especially useful for testing immunotherapy and precision medicine for cancers. Therefore, the identification of biomarkers and combination therapies for canine cancer may not only improve veterinary practice but also benefit human clinical oncology.

Serum PGE_2_, IL-12p40, IL-8, MCP-1, and SCF concentrations were higher in dogs with OMM than healthy controls, providing clues to the immunological changes associated with tumorigenesis and progression of OMM, including resistance to endogenous antitumor mechanisms. These findings are consistent with previous reports showing elevated serum IL-12p40 in osteosarcoma^42^, IL-8 in mammary tumor and osteosarcoma^42–44^, and MCP-1 in lymphoma, histiocytic sarcoma, urothelial carcinoma, and malignant melanoma^45–49^ compared to healthy dogs. Upregulation of these factors may reflect immunological responses in the tumor microenvironment, but the cellular and locoregional sources of these factors remain to be investigated.

Higher serum MCP-1 and VEGF-A concentrations were associated with poorer OS in OMM dogs receiving c4G12 therapy, in accordance with previous reports on human melanoma patients under ICI therapy^21,50^. MCP-1 is an inducible chemokine regulating macrophage infiltration into tumors^51^. In humans, both primary and metastatic melanomas, but not normal skin, express MCP-1^52^, and antagonism of its receptor CCR2 enhanced the therapeutic efficacy of anti-PD-1 antibody in a murine melanoma model^53^. Considering the protumor propensity of tumor-associated macrophages^54^, it is plausible that canine OMM tissues overexpress MCP-1 and thereby attract monocytes/macrophages, which in turn create an immunosuppressive microenvironment and confer resistance to anti-PD-L1 therapy. Similarly, VEGF-A is also immunosuppressive in the tumor microenvironment^55^ in addition to acting as the primary driver of tumor angiogenesis. Numerous human clinical studies have revealed that anti-VEGF agents in combination with ICIs show promising efficacy against neoplasms such as renal cell carcinoma and hepatocellular carcinoma^56^. Although a similar immunosuppressive activity has not been demonstrated in dogs, VEGF overexpression has been demonstrated by immunohistochemistry in canine OMM^57^. Thus, combined VEGF targeting and ICI therapy warrants further investigation. The prolonged OS observed in OMM subgroups exhibiting high serum IL-2, IL-12p40, and SCF concentrations is also suggestive of a strong association between antitumor immune response and clinical outcome. Both IL-2 and IL-12 contribute to T cell activation, so the baseline serum concentrations of these cytokines may indicate antitumor immune status in OMM dogs. In contrast, the mechanistic implications of elevated SCF, a growth factor/cytokine involved in hematopoiesis through binding to its receptor c-Kit^58^, are still unclear as relevance of SCF to antitumor immunity has not been established. As a limitation of the survival analyses, only a small number of dogs (*n* = 27) were included, allowing only exploratory univariate analyses. Because the univariate analysis may contain multiple biases (e.g., breeds, age, and sex), the results should carefully be interpreted. Multivariate analyses of a larger cohort and validation in an independent cohort are needed to determine which of these aforementioned factors can be used as predictive biomarkers in veterinary practice. In addition, further investigation is needed to clarify whether these potential biomarkers are specific to anti-PD-L1 therapy or also applicable to other treatment modalities of canine OMM.

Prostaglandin E_2_ is a well-known mediator of acute local inflammation^38^. However, PGE_2_ also serves as an immunosuppressive factor in late or chronic phases by reducing T cell, NK cell, and dendritic cell functions and by promoting regulatory T cell development^38^. The PGE_2_ biosynthetic enzyme COX-2 is often upregulated in premalignant and malignant tissues^59^, and promotes cancer cell survival, metastasis, and angiogenesis^59,60^ as well as immune evasion^61^. Therefore, pharmacological targeting of COX-2 is considered a promising approach for cancer prevention and therapy^59^. Recently, crosstalk between the PD-1/PD-L1 and COX-2/PGE_2_ pathways was reported^28,62,63^. In mouse tumor models, COX inhibition by aspirin or celecoxib enhanced the efficacy of anti-PD-1 antibody^28^, implying that inhibition of the COX-2/PGE_2_ pathway could be a useful adjuvant to ICI treatment. Indeed, we previously reported that dual blockade of these pathways enhanced ICI therapeutic efficacy in cattle with bovine leukemia virus infection and in a mouse lymphoma model^64,65^, suggesting that this combination strategy is applicable across animal species and diseases.

To our knowledge, this is the first report to demonstrate that, in any animal species, baseline serum PGE_2_ concentration is associated with clinical outcome of ICI therapy and that PGE_2_ is a suppressor of canine immune cell activation. We also demonstrate that COX-2 inhibition plus anti-PD-L1 antibody treatment enhances immune cell activation, further supporting the potential of this combination therapy for treatment of canine cancers including OMM. Given that COX-2 overexpression is a common feature of various cancer types, that COX-2 inhibitors are already widely used in clinical practice with well-known and manageable side effects profiles, and that COX-2 activity is involved in malignant phenotype independent of immune suppression, we suggest that COX-2 inhibitors are broadly applicable, safe, and effective drugs for ICI combination therapy. Nonetheless, careful attention should be paid to possible side effects of the combination treatment. Treatment-related adverse events of c4G12 included pneumonitis, elevated liver enzymes and lipase, vomiting, and diarrhea^16^, while meloxicam treatment is reported to be associated with gastrointestinal (vomiting, diarrhea, and ulceration), urinary (azotemia and renal failure), hepatic (elevated liver enzymes), and dermatologic (pruritus) abnormalities^66^. Because gastrointestinal and hepatic toxicities are suggested for both treatments, frequency and severity of these events must be monitored in particular in future studies on the combination therapy.

In conclusion, we have identified several serum factors including PGE_2_ as potential predictive biomarkers of prolonged survival in dogs with OMM receiving anti-PD-L1 therapy. Further, we identified the COX-2/PGE_2_ axis as a potential target to enhance the efficacy of ICI therapy in dogs. The overall consistency with human biomarker analyses and preclinical studies suggests that dogs could be a clinically relevant, large animal model for ICI therapy. The clinical study of this combination therapy for canine cancer is now in progress in our veterinary hospital.

## Supplementary Information

Supplementary Tables.
