# Supplementary material for: Exploration of serum biomarkers in dogs with malignant melanoma receiving anti-PD-L1 therapy and potential of COX-2 inhibition for combination therapy
Source: Sci Rep. 2022 Jun 3;12:9265. doi: 10.1038/s41598-022-13484-8 (PMC9166720; doi:10.1038/s41598-022-13484-8)
Supplement: Supplementary file 1 — Supplementary Tables. [file 41598_2022_13484_MOESM1_ESM.pdf]

Exploration of serum biomarkers in dogs with malignant melanoma receiving anti-PD-L1 therapy and potential of COX-2 inhibition for combination therapy

Naoya Maekawa, Satoru Konnai*, Yumie Asano, Yamato Sajiki, Tatsuya Deguchi, Tomohiro Okagawa, Kei Watari, Hiroto Takeuchi, Satoshi Takagi, Kenji Hosoya, Sangho Kim, Hiroshi Ohta, Yukinari Kato, Yasuhiko Suzuki, Shiro Murata, Kazuhiko Ohashi

*Corresponding author

Satoru Konnai

E-mail: konnai@vetmed.hokudai.ac.jp

Supplementary Table 1. Baseline characteristics and treatment outcome of c4G12-treated dogs.

Dog No.	Breed	Sex	Age (year)	Overall survival (days)	Tumor response*
1	Pug	Male, castrated	11	220	No
2	Miniature dachshund	Male, castrated	14	81	No
3	Golden retriever	Male, castrated	10	86	No
4	Miniature dachshund	Male, castrated	14	88	No
5	Beagle	Female, spayed	11	362	Yes
6	American cocker spaniel	Male, castrated	13	108	No
7	Yorkshire terrier	Male	14	44	No
8	Kaninchen dachshund	Female, spayed	13	35**	No
9	Miniature dachshund	Male	13	127	No
10	Pomeranian	Male	12	168†	Yes
11	Mix	Female	12	180	No
12	Pekingese	Male, castrated	8	385	Yes
13	Mix	Male, castrated	11	61‡	No
14	Miniature dachshund	Male	13	204	No
15	Miniature dachshund	Female	15	44	No
16	Labrador retriever	Female	11	98	No
17	Toy poodle	Female, spayed	13	58	No
18	Miniature dachshund	Female, spayed	15	518**	Yes
19	Miniature dachshund	Male	14	118	No
20	Labrador retriever	Male	12	24	No
21	Toy poodle	Male	16	105**	No
22	Chihuahua	Female, spayed	13	185	No
23	Welsh Corgi	Male, castrated	10	63	No
24	Papillon	Male, castrated	14	42**	No
25	Flat-coated retriever	Female, spayed	8	138	No
26	Golden retriever	Male, castrated	14	154**	Yes
27	Miniature dachshund	Male, castrated	14	53	No

*Tumor response in at least one detectable lesion as evidenced by diagnostic imaging [16].
**Censored data due to loss to follow-up/withdrawal of consent or the end of study period.
†Died from chronic kidney disease.
‡Euthanized due to disease progression.

Supplementary Table 2. Serum concentrations of each factor at baseline of c4G12 treatment.

Dog No.	PGE_2	IFN-γ	IL-2	IL-6	IL-10	IL-12p40	TNF-α	IL-8	MCP-1	NGF-β	SCF	VEGF-A
1	10.2	< LLOQ	< LLOQ	< LLOQ	< LLOQ	723.0	< LLOQ	2825.6	132.3	< LLOQ	109.8	6.7
2	6.7	< LLOQ	47.3	41.0	42.0	1225.9	3.7	2392.3	163.2	17.7	57.3	33.6
3	9.4	< LLOQ	< LLOQ	< LLOQ	< LLOQ	790.7	< LLOQ	1711.5	154.4	< LLOQ	86.0	26.7
4	11.8	< LLOQ	< LLOQ	< LLOQ	< LLOQ	227.2	< LLOQ	3204.9	68.4	< LLOQ	16.6	< LLOQ
5	2.8	< LLOQ	58.3	313.0	< LLOQ	1009.7	< LLOQ	1154.7	88.5	< LLOQ	303.0	< LLOQ
6	9.7	< LLOQ	27.5	45.8	< LLOQ	1338.4	< LLOQ	4383.6	121.4	< LLOQ	226.2	28.5
7	7.6	< LLOQ	< LLOQ	< LLOQ	< LLOQ	243.8	< LLOQ	1462.5	95.0	< LLOQ	13.0	5.5
8	4.0	< LLOQ	< LLOQ	< LLOQ	71.9	1005.1	< LLOQ	1236.2	304.7	79.0	43.5	< LLOQ
9	10.4	< LLOQ	< LLOQ	< LLOQ	< LLOQ	589.2	< LLOQ	1519.0	176.9	< LLOQ	47.1	14.3
10	7.4	< LLOQ	574.0	560.0	1130.6	3026.7	< LLOQ	6004.0	115.2	< LLOQ	390.8	22.1
11	6.5	< LLOQ	208.8	153.5	29.3	1757.0	< LLOQ	1980.8	172.7	< LLOQ	397.0	7.3
12	11.1	< LLOQ	33.0	< LLOQ	< LLOQ	651.9	4.7	2701.9	110.4	< LLOQ	47.3	9.6
13	6.0	< LLOQ	69.2	35.7	< LLOQ	1494.4	< LLOQ	1410.8	69.5	< LLOQ	130.8	7.5
14	3.4	41.6	< LLOQ	< LLOQ	45.0	388.5	< LLOQ	3469.0	17.6	< LLOQ	40.8	18.2
15	9.8	< LLOQ	< LLOQ	< LLOQ	19.5	1104.2	< LLOQ	2731.4	68.1	< LLOQ	80.2	22.3
16	7.7	48.0	12.1	19.1	10.3	688.7	< LLOQ	1832.3	208.2	< LLOQ	116.8	15.4
17	5.9	3.2	< LLOQ	< LLOQ	< LLOQ	267.2	< LLOQ	945.5	238.0	< LLOQ	27.3	< LLOQ
18	6.5	< LLOQ	274.7	142.4	18.4	3004.6	< LLOQ	2483.0	48.7	28.0	190.1	17.9
19	9.4	< LLOQ	< LLOQ	< LLOQ	< LLOQ	189.1	< LLOQ	2221.9	110.5	< LLOQ	28.6	12.0
20	7.2	10.4	11.4	< LLOQ	11.6	1293.5	< LLOQ	1843.4	281.3	< LLOQ	80.0	26.7
21	2.4	52.8	< LLOQ	< LLOQ	< LLOQ	621.2	< LLOQ	1302.4	89.2	< LLOQ	116.7	13.0
22	4.5	< LLOQ	< LLOQ	< LLOQ	< LLOQ	641.9	< LLOQ	3235.1	220.5	< LLOQ	87.7	44.1
23	13.6	< LLOQ	< LLOQ	10.7	< LLOQ	402.0	< LLOQ	4474.4	172.7	< LLOQ	69.0	15.2
24	6.4	< LLOQ	< LLOQ	< LLOQ	< LLOQ	610.0	< LLOQ	1023.8	57.7	< LLOQ	58.1	6.8
25	4.0	< LLOQ	17.7	< LLOQ	< LLOQ	972.2	< LLOQ	1069.4	39.6	< LLOQ	304.0	< LLOQ
26	3.1	< LLOQ	< LLOQ	< LLOQ	84.6	697.0	37.9	2007.0	81.4	185.8	125.0	19.9
27	11.0	< LLOQ	14.1	18.0	< LLOQ	1474.0	12.4	1541.7	792.5	84.2	103.4	32.5

PGE_2, ng/mL; others, pg/mL.
LLOQ, lower limit of quantification.

Supplementary Table 3. Characteristics of dogs with tumors used in plasma PGE_2 measurement.

Breed	Sex	Age (year)	Pathology	Tumor site
Miniature dachshund	Female, spayed	12	Malignant melanoma	Mandible
Mix	Male, castrated	13	Malignant melanoma	Mandible
Shiba Inu	Male	14	Malignant melanoma	Mandible
Shiba Inu	Female, spayed	16	Malignant melanoma	Mandible
Labrador retriever	Female, spayed	13	Osteosarcoma	Vagina
Beagle	Female, spayed	15	Osteosarcoma	Rib
Standard poodle	Male	9	Osteochondrosarcoma	Malar
Labrador retriever	Male	14	Chondrosarcoma	Nasal cavity
Golden retriever	Female, spayed	11	Hemangiosarcoma	Spleen
Mix	Female, spayed	12	Hemangiosarcoma	Spleen
Leonberger	Female	10	Hemangiosarcoma	Spleen
Japanese spits	Male, castrated	12	Lymphoma	Small intestine
Miniature dachshund	Female, spayed	12	Lymphoma	Small intestine
West Highland white terrier	Female, spayed	15	Lymphoma	Small intestine
Mix	Female, spayed	11	Meningioma	Brain
Labrador retriever	Male	12	Meningioma	Brain
Toy poodle	Male, castrated	11	Prostate adenocarcinoma	Prostate
Jack Russel terrier	Male, castrated	9	Hepatocellular adenoma	Liver
Miniature dachshund	Male	13	Pheochromocytoma	Adrenal gland
Golden retriever	Female	10	Thymoma	Thymus
Shih tzu	Male, castrated	13	Sebaceous gland adenoma	Skin
